# Stem Cell Therapy Enhances Motor Activity of Triceps Surae Muscle in Mice with Hereditary Peripheral Neuropathy

**DOI:** 10.3390/ijms222112026

**Published:** 2021-11-06

**Authors:** Iryna Govbakh, Vitalii Kyryk, Alina Ustymenko, Volodymyr Rubtsov, Oleg Tsupykov, Nataliya V. Bulgakova, Danylo O. Zavodovskiy, Inna Sokolowska, Andriy Maznychenko

**Affiliations:** 1 Department of General Practice-Family Medicine, Kharkiv Medical Academy of Postgraduate Education, 61000 Kharkiv, Ukraine; irynagovbakh@gmail.com; 2 Cell and Tissue Technologies Department, State Institute of Genetic and Regenerative Medicine NAMS of Ukraine, 04114 Kyiv, Ukraine; biomedpost@gmail.com (V.K.); alina.n.ustymenko@gmail.com (A.U.); tsupykov@gmail.com (O.T.); 3 Laboratory of Pathophysiology and Immunology, D. F. Chebotarev State Institute of Gerontology NAMS of Ukraine, 04114 Kyiv, Ukraine; 4 Department of Cytology, Histology and Reproductive Medicine, Educational and Scientific Institute of Biology and Medicine, Taras Shevchenko National University of Kyiv, 03127 Kyiv, Ukraine; rubtsov228volodymyr@gmail.com; 5 Department of Cytology, Bogomoletz Institute of Physiology NAS of Ukraine, 01024 Kyiv, Ukraine; 6 Department of Movement Physiology, Bogomoletz Institute of Physiology NAS of Ukraine, 01024 Kyiv, Ukraine; bulgakova@biph.kiev.ua (N.V.B.); lab@univ.kiev.ua (D.O.Z.); 7 Department of Physical Education, Gdansk University of Physical Education and Sport, 80-336 Gdansk, Poland; inna.sokolowska@awf.gda.pl

**Keywords:** stem cells, hereditary peripheral neuropathy, muscle contraction, electrical stimulation, mice

## Abstract

Impaired motor and sensory functions are the main features of Charcot–Marie–Tooth disease. Mesenchymal stem cell (MSCs) therapy is one of the possible treatments for this disease. It was assumed that MSCs therapy can improve the contractile properties of the triceps surae (TS) muscles in mice with hereditary peripheral neuropathy. Murine adipose-derived mesenchymal stromal cells (AD-MSCs) were obtained for transplantation into TS muscles of *FVB-C-Tg(GFPU)5Nagy/J* mice. Three months after AD-MSCs transplantation, animals were subjected to electrophysiological investigations. Parameters of TS muscle tension after intermittent high frequency electrical sciatic nerve stimulations were analyzed. It was found that force of TS muscle tension contraction in animals after AD-MSCs treatment was two-time higher than in untreated mice. Normalized values of force muscle contraction in different phases of electrical stimulation were 0.3 ± 0.01 vs. 0.18 ± 0.01 and 0.26 ± 0.03 vs. 0.13 ± 0.03 for treated and untreated animals, respectively. It is assumed that the two-fold increase in TS muscle strength was caused by stem cell therapy. Apparently, AD-MSCs therapy can promote nerve regeneration and partial restoration of muscle function, and thus can be a potential therapeutic agent for the treatment of peripheral neuropathies.

## 1. Introduction

One of the most common hereditary functional disorders of the peripheral nervous system (PNS) is Charcot–Marie–Tooth disease of type 1A (CMT1A) [1,2,3]. Point mutations in the *PMP22* gene, as well as DNA duplication in the region of this gene, are some of the main causes of the disease development. Both of these reasons are associated with impaired peripheral myelination and, depending on the degree of damage, can lead to slow down conduction in nerve fibers, impaired mobility of the limbs and, later, to its deformation [4,5,6,7].

For the purpose of research and diagnosis of CMT1A disease, neurophysiological methods are often used to determine the speed of conduction in a nerve or muscle [6,8], as well as various behavioral tests [7,9]. In addition, some researchers use gene therapy to treat CMT1A. This therapy consists of delivering a functional copy of the gene that causes the disease. In a mouse model CMT1A, virus was used to deliver a functional version of the *NT-3* gene. This gene is involved in supporting the viability of Schwann cells, which maintain the protective myelin sheath that surrounds the nerves. It has been shown that such type of therapy to promote nerve regeneration [10].

Despite the large number of studies for this topic with a fairly detailed description of the main mechanisms of the disease, there is still no effective treatment for CMT1A [11]. The treatment of this disease was symptomatic and consisted mainly of methods of physical rehabilitation [12,13].

In recent times, stem cells have been used to treat experimental CMT1A. Stem cell therapy is seen as a promising treatment for peripheral neuropathies. Cell therapy using mesenchymal stromal cell (MSCs) is the most clinically advanced form of stem cell therapy, second to hematopoietic stem cell transplants [14]. MSCs are able to differentiate into various tissue-specific cell types [15]. It was demonstrated that MSCs secrete a plethora of molecules, such as chemokines, cytokines, growth factors and anti-apoptotic factors with neuroprotective activities. For example, it has been shown that MSCs from adipose tissue produce anti-apoptotic factors such as HFG, VEGF and IGF in response to pro-apoptotic mediators [16]. Furthermore, adipose-derived mesenchymal stromal cells (AD-MSCs) represent a valuable source of stem cells because of their relative abundance, ease of isolation, and expandability [17]. Recently, in the morphological study was shown that in mice with sciatic nerve neuropathy induce both thickening of the myelin sheath and increase in the number of lamellae, after AD-MSCs transplantation. In this way, in mice with hereditary peripheral neuropathy, AD-MSCs transplantation has a protective effect on the ultrastructural features of the sciatic nerve and inhibits the process of axonal demyelination [18].

Thus, taken into account the positive effect of stem cell therapy on the nerve regeneration, we supposed that this type of treatment can improve the motor activity of the muscles in mice with hereditary peripheral neuropathy. Thus, the purpose of this study was to reveal effect of stem cell therapy on the triceps surae (TS) muscle contractile properties.

## 2. Results

**Figure 1:**
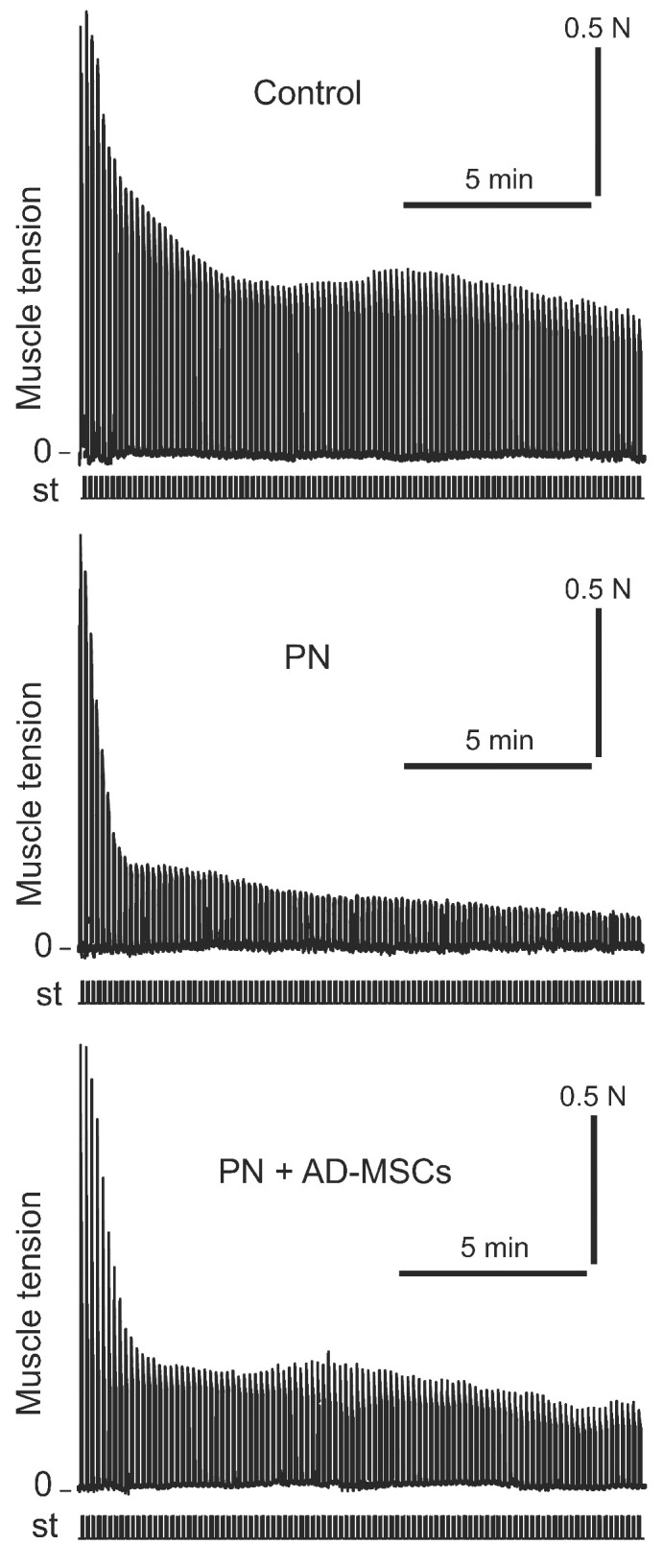
Examples of registration protocol of the triceps surae muscle contractions force of one Control animal, one mouse with peripheral neuropathy (PN) and one treated animal with peripheral neuropathy (PN+AD-MSCs). N—muscle force (Newton), st—stimulation mark.

**Figure 2:**
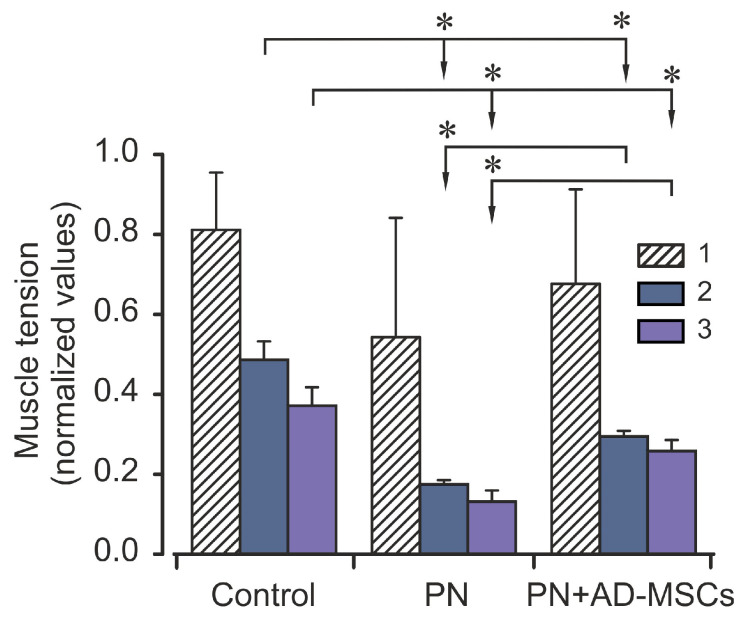
Averaged characteristics (mean ± SD) of normalized values of the triceps surae muscle strength during different phases of the electrical stimulation of Control animals, mice with peripheral neuropathy (PN) and treated animals with peripheral neuropathy (PN+AD-MSCs). Asterisks (*) designated significant differences (*p* < 0.05) in the muscle strength during the same stimulation phases between animals of all groups. 1, 2 and 3 –phases of the electrical stimulations (0–100 s, 101–200 s and 201–900 s, respectively).

Intermittent high-frequency stimulation of sciatic nerve induced distinctive contraction pattern of triceps surae muscle in animals of all groups. It was expressed in a sharp decrease in the strength of muscle contraction during (approximately) the first 100 s, then was changed by a phase of moderate contraction (which occurred between 100 and 200 s), and then was followed by a gradual decrease in muscle effort until the end of stimulation. Examples of this pattern have been shown in Figure 1. Nevertheless, although the patterns of muscle contraction were similar, but the strengths of muscle contractions during these phases in different animal groups were differ significantly (Figure 2). It should be noted that the changes of the TS muscle force reaction during first phase among mice of all groups did not differ significantly (*p* > 0.05), however, muscle power reaction for the period of second and third phases were significantly different (*p* < 0.05). In comparison with the control (2nd phase), the amplitude of force muscle contraction in animals of PN group was 65% less, whereas in animals which were subjected stem cell therapy (PN+AD-MSCs group) this parameter was decreasing by 40%. During the 3rd phase of muscle stimulation these indicators were 65% and 30% less (groups 2 and 3, respectively) compared with the control. Normalized values of the PN+AD-MSCs and PN mice during second phase were 0.3 ± 0.01 vs. 0.18 ± 0.01 and 0.26 ± 0.03 vs. 0.13 ± 0.03, respectively. Bonferroni *post hoc* analysis was shown a significant effect of stem cell treatment on muscle force contraction development as compared to PN animals (F_1,10_ = 288.0, *p* < 0.001 and F_1,10_ = 61.7, *p* < 0.001 during the 2nd and 3rd phase of stimulation, respectively). Differences between control and PN mice as well as control and PN+AD-MSCs animals were F_1,10_=262.9, *p* < 0.001 and F_1,10_=95.97, *p* < 0.001 (2nd phase), and F_1,10_ = 118.6, *p* < 0.001 and F_1,10_ = 26.45, *p* < 0.001 (3rd phase), respectively.

**Figure 3:**
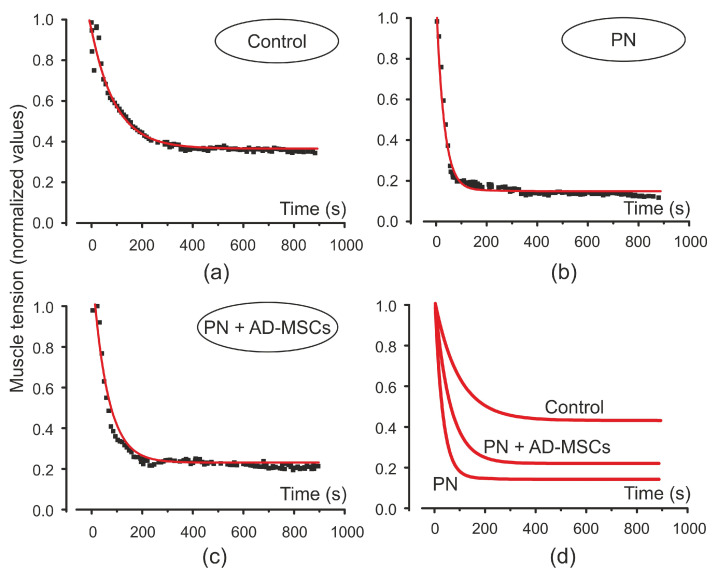
Amplitude values of the triceps surae muscle force induced by electrical stimulation at the beginning of single tetanic contractions (squares) and its exponential approximation (red curve) in control animals (**a**), mice with peripheral neuropathy (PN) (**b**) and treated animals with peripheral neuropathy (PN+AD-MSCs) (**c**). Exponential curves for all animals’ groups shown on (**d**).

As mentioned above, after electrical stimulation force of the isometric muscle contraction after its fast decrease was changed by a gradual decrease. Such changes in the level of the muscle tension during stimulation are quantitatively described by an exponential approximation (Figure 3). The time constants (τ) of the approximation for isometric contractions were 30.7, 60.03 and 95.4 for PN, PN+AD-MSCs and control animal groups, respectively. It can be seen that τ for PN+AD-MSCs group was two times more than for the PN mice although it was statistically different from the control. The time constant τ is well described by the value of its amplitude, it was 0.94, 0.97 and 0.58 (for the same sequence of groups). Although the amplitude of the process in the PN group did not significantly differ from those that were treated, the time constant τ showed that the rate of decrease in muscle contraction was two times lower in the PN+AD-MSCs group.

## 3. Discussion

It is known that MSCs therapy of neurological disorders is still at a relatively early phase of clinical application, and there are not any MSCs related registered products for neurological disorders [19]. However, recent studies showed that cell therapies using mesenchymal stem cells represent an emerging strategy for neurological lesions and disorders [19,20]. In addition, it has been shown that MSCs may improve the neurological disease in the following possible ways: homing to the injured site, paracrine factors, and immunomodulation [21]. Numerous studies have demonstrated the paracrine activity of MSCs via the secretion of a wide range of neurotrophic and growth factors (VEGF, BDNF, NGF, GDNF, CNTF), which are able to promote axonal growth and remyelination [22]. In addition, MSC secretome includes immunoregulatory factors (IL-6, IL-1Ra, IL-10, IL-13, PGE2) [23]. It has also been shown that the MSC-derived exosome have potentially therapeutic effectors, and contains growth factors and cytokines, signaling lipids, mRNAs, and regulatory miRNAs [24].

In the study we investigated changes in the TS muscle tension induced by electrical stimulation in mice with hereditary peripheral neuropathy. It was established that application of the MSCs in *B6.Cg-Tg(PMP22)C3Fbas/J* mice induce improvement of muscle contraction characteristics. It has been found that the speed of reduction of muscle contraction force in treated animals was twice as slow as in the mice that did not receive this therapy. In addition, animals of the 3rd group can hold 2-fold higher level of muscle force contraction during the stimulation in comparison with the PN mice. Apparently, these changes are associated with a partial recovery of the function of the nerve processes.

Our finding is in accordance with the study by Park S. et al., who showed improvement of neuromuscular function in a mouse model of Charcot-Marie-Tooth disease Type 1A after transplantation of Schwann-like cells differentiated from human tonsil-derived stem cells [25]. Studies show that trophic molecules secreted by MSCs work in a complementary way during nerve regeneration, in order to promote the survival and outgrowth of axons and Schwann cells [26].

Schwann cells are a key component of nerve regeneration. It has previously been shown that stem cells can differentiate into neurons that will generate new axons, or Schwann cells (SCs), surrounding demyelinated axons [27]. It has also been shown that MSCs can improve myelin formation and regeneration of damaged peripheral nerves [28].

Mesenchymal stromal cells from different tissues, such as adipose-derived stem cells (AD-MSCs) [29], bone marrow-derived stem cells (BM-MSCs) [30], and umbilical cord-derived MSCs (UC-MSCs) [31] were used to promote peripheral nerve regeneration.

**Figure 4:**
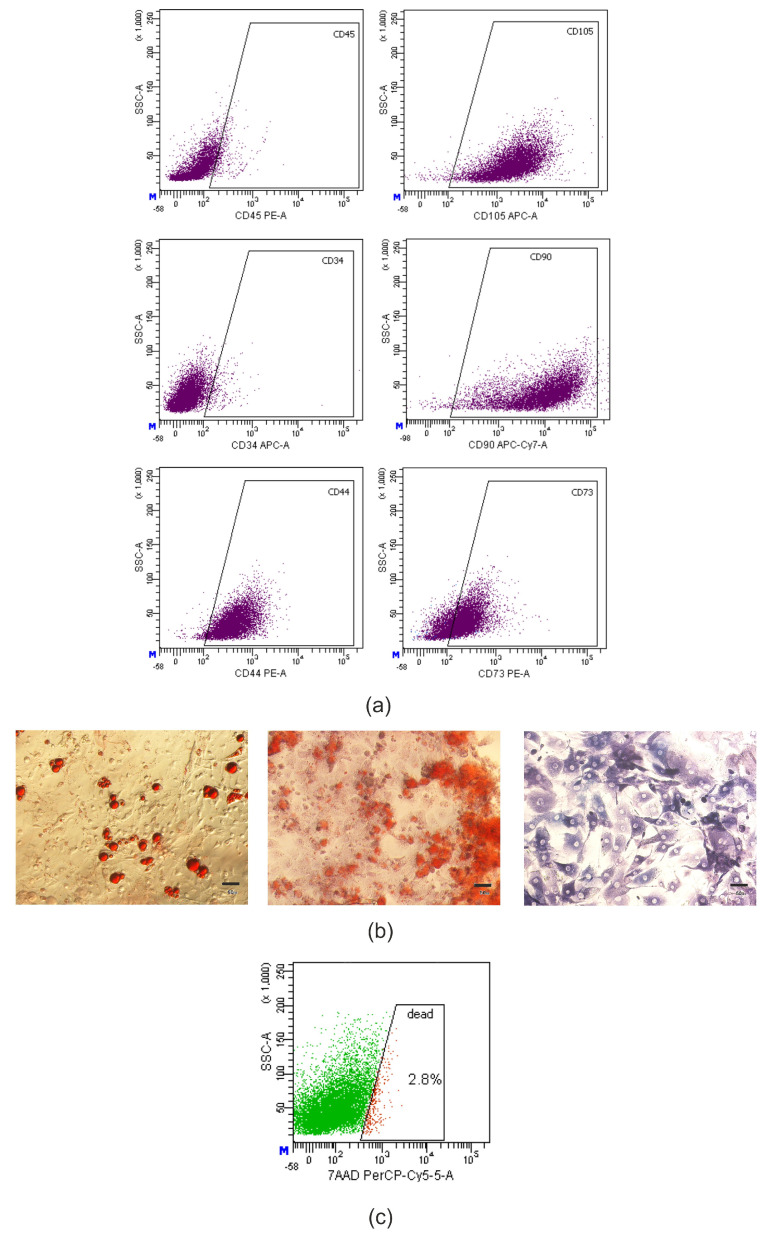
Murine AD-MSCs cultures met the minimal criteria to define MMSCs. (**a**) dot-plot histograms of CD44, CD73, CD90, CD105, CD34 and CD45 markers expression in the culture of murine AD-MSCs according to flow cytometry, 2nd passage. (**b**) photomicrographs of murine AD-MSCs cultures after directed adipogenic (left) and osteogenic (middle and right) differentiation on the 21st day of culturing. Lipid droplets stained with Oil Red O (red); calcium deposits in mineralized extracellular matrix stained with Alizarin Red S (pink); alkaline phosphatase stained with BCIP/NBT (violet); light microscopy. Scale bar = 50 μm. (**c**) dot-plot histogram of AD-MSCs viability after thawing, 7-aminoactinomycin D (7-AAD) staining.

Adipose-derived mesenchymal stromal cells have been shown to have a number of advantages: it can be obtained in relatively large quantities; AD-MSCs escape immune system surveillance; AD-MSCs produce adhesion molecules that enable their extravasation into the damaged PNS [32,33,34]. Another significant advantage of these cells is that AD-MSCs can retain high degrees of viability after thawing, as we have also shown in our experiments (see Materials and Methods, Figure 4c). This is very important for cryopreservation and storage of AD-MSCs in cryobanks for their further use in cell therapy.

Zhou et al., [35] found that AD-MSCs with autologous nerve grafts and acellular grafts to cause regeneration through a sciatic nerve defect in rats. Other studies have shown that implantation of SCs derived from AD-MSCs showed improved musculoskeletal function [36] as well as decreased neuronal damage [37]. Earlier in the morphological study [18] it was shown that transplantation of AD-MSCs into mice with peripheral neuropathy of the sciatic nerve causes a thickening of the myelin sheath of axons and an increase in the number of lamellae. These results are consistent with reports of enhanced sciatic nerve regeneration by restoring the myelin sheath and axons of peripheral nerves in mice with peripheral neuropathy after MSCs transplantation [25]. The crosstalk with recipient’s glial cells may explain how a limited number of transplanted AD-MSCs may amplify their neuroprotective effects [29].

As is known motor and sensory impairments are main outcome of Charcot-Marie-Tooth disease, and the main goal in this disease treatment is the regeneration of cell viability based on improving myelin and axon interaction [27]. As shown above, MSCs therapy can promote nerve regeneration, and thus be a potential therapeutic agent for the treatment of peripheral neuropathies.

## 4. Materials and Methods

### 4.1. Experimental Animals

All animals were maintained under controlled light and environment (12:12 h light/dark cycle, 24 ± 1 °C), and provided with water and food pellets *ad libitum*. All procedures complied with the ARRIVE guidelines. The use of the animals was approved by the Committee for Biomedical Ethics of the Institute (#2/22, 26 February 2020) and performed in accordance with the European Union Directive of 22 September 2010 (2010/63/EU) for the protection of animals used for scientific purposes.

Three groups of mice (aged 7 months) both sexes with a body weight of 23–29 g were used in the study. Group I–control healthy animals of the *C57BL* strain (*n* = 6). Group II–transgenic *B6.Cg-Tg(PMP22)C3Fbas/J* mice with peripheral neuropathy (PN) (*n* = 6). Group III–*B6.Cg-Tg(PMP22)C3Fbas/J* mice (*n* = 6) after transplantation of adipose-derived stem cells (PN+AD-MSCs). *B6.Cg-Tg(PMP22)C3Fbas/J* mice were obtained from The Jackson Laboratory (USA). To obtain AD-MSCs, we used male *FVB-C-Tg(GFPU)5Nagy/J* mice, transgenic for green fluorescent protein (GFP), aged 5 months (*n* = 8). The mice were kindly provided by the European Molecular Biology Laboratory (Monterotondo, Italy).

### 4.2. Stem Cells Isolation and Characterization

#### 4.2.1. Adipose-Derived Mesenchymal Stromal Cells Isolation and Culture

Murine AD-MSCs cultures were obtained and characterized as described previously [9]. The *FVB-C-Tg(GFPU)5Nagy/J* mice were euthanized by cervical dislocation under the anesthesia with 2.5% solution of 2,2,2-tribromethanol (avertin at a dose 400 mg/kg). Under sterile conditions, subcutaneous adipose tissue was isolated, minced with scissors into 1 mm^3^ pieces in DMEM/F12 medium (“Sigma–Aldrich”, St. Louis, MO, USA) and incubated in 0.1% solution of collagenase type IA (“Sigma–Aldrich”, St. Louis, MO, USA) for 90 min at 37 °C with constant stirring on a shaker at 100 rpm. The resulting cell suspension was washed in 10 mL DMEM medium (“Sigma–Aldrich”, St. Louis, MO, USA) by centrifugation at 300× *g* for 5 min. The supernatant with mature adipocytes and debris was discarded and pellet passed through a sterile cell strainer with a pore diameter of 100 μm (“Greiner bio-one”, Kremsmünster, Austria). Cells of the stromal-vascular fraction were cultured in a CO_2_ incubator in humidified atmosphere with 5% CO_2_ at a temperature of +37 °C in complete nutrient medium DMEM-LG (“Sigma–Aldrich”, St. Louis, MO, USA) supplemented with 15% fetal bovine serum (FBS) (“HyClone” Laboratories Inc., South Logan, UT, USA), penicillin 100 U/mL, streptomycin 100 μg/mL (“Sigma–Aldrich”, St. Louis, MO, USA), 1:100 nonessential amino acids (“Sigma–Aldrich”, St. Louis, MO, USA). The nutrient medium was replaced in 3 days. Cells were subcultured to achieve 80% monolayer confluency using 0.25% trypsin solution (“Sigma–Aldrich”, St. Louis, MO, USA) and 0.02% versene (“Bio-Test Laboratory”, Kyiv, Ukraine).

On the 2nd passage, immunophenotyping and directed differentiation towards osteogenic and adipogenic lineages of the obtained cultures were performed according to standard methods, as described previously [38]. Cells were analyzed by flow cytometry with BD FACSAria cell sorter (“Becton Dickinson”, Franklin Lakes, NJ, USA) using anti-mouse monoclonal antibodies: CD90 APC-Cy7 (“BD Biosciences”, cat. no. 561401, Franklin Lakes, NJ, USA), CD105 APC (“Invitrogen”, cat. no. 17-1051-82, Carlsbad, CA, USA), CD73 PE (“BD Biosciences”, cat. no. 550741, Franklin Lakes, NJ, USA), CD44 PE (“BD Biosciences”, cat. no. 553134, Franklin Lakes, NJ, USA), CD45 PE (“Thermo Fisher Scientific”, cat. no. MA1-10233, Waltham, MA, USA), CD34 Alexa Fluor 647 (“BD Biosciences”, cat. no. 560230, Franklin Lakes, NJ, USA).

There was identified high expression of specific mesenchymal markers CD44, CD73, CD90, CD105, and, at the same time, low level of hematopoietic markers CD34, CD45 (Figure 4a).

At the directed adipogenic differentiation, there were determined lipid droplets in a cytoplasm of cells by staining with Oil Red O (“Sigma–Aldrich”, St. Louis, MO, USA). At osteogenic differentiation, there was a deposition of calcium salts in an extracellular matrix using Alizarin Red S (“Sigma–Aldrich”, St. Louis, MO, USA) and positive staining for alkaline phosphatase using 5-bromo-4-chloro-3-indolyl-phosphate/nitro blue tetrazolium (BCIP/NBT) (“Sigma–Aldrich”, St. Louis, MO, USA) (Figure 4b).

Thus, the obtained cultures met the minimal criteria to define AD-MSCs in terms of morphology, adhesive properties, immunophenotype and potential for directed differentiation [39,40].

The cells of the 2nd passage were resuspended at a concentration of 1 × 10^6^ cells/mL in a cryopreservation medium consisting of 90% FBS and 10% dimethyl sulfoxide (“Sigma–Aldrich”, St. Louis, MO, USA), frozen to −80 °C in containers CoolCell^®^ (“Corning”, Corning, NY, USA) and stored in cryostorage with liquid nitrogen at −196 °C.

#### 4.2.2. Transplantation of AD-MSCs

Prior to transplantation, the cells were thawed in a water bath at 37 °C, washed by centrifugation at 350× *g* for 10 min in 10 mL of DMEM/F12 medium supplemented with 10% FBS, and resuspended in phosphate buffered saline (PBS) (“HyClone” Laboratories Inc., South Logan, UT, USA). Viability of thawed cells analyzed by flow cytometry using staining with 7-aminoactinomycin D (7-AAD) was 97.2% (Figure 4c). The *B6.Cg-Tg(PMP22)C3Fbas/J* mice with peripheral neuropathy were transplanted with 0.5 × 10^6^ GFP-positive AD-MSCs in 50 μL of PBS intramuscularly in *m. gastrocnemius* on both sides under intraperitoneal (i.p.) anesthesia (calypsol + xylazine, 75 and 2 mg/kg (“Pfizer”, New York, NY, USA) body weight, respectively). The animals of group II were injected with 50 μL of PBS without cells intramuscularly in *m. gastrocnemius* on both sides.

Three months after AD-MSCs transplantation, animals were subjected to electrophysiological investigations.

### 4.3. Electrophysiological Experiment

The animals of all groups were anaesthetized (i.p.) with ketamine (100 mg/kg “Pfizer”, USA) combined with xylazine (10 mg/kg, “Interchemie”, Venray, The Netherland). The left TS muscles were separated from the surrounding tissue, and their tendons were detached at the distal insertions. The sciatic nerve was separated from the tissue and cut proximally. This nerve was mounted on a bipolar platinum wire electrode for electrical stimulation. The hindlimb muscles and nerves were covered with paraffin oil in a pool formed from skin flaps. The TS muscle was connected via the Achilles tendon to the servo-control muscle puller. The muscle tension was measured by semi-conductor strain gauge resistors glued on a stiff steel beam mounted on the moving part of a linear motor.

To induce muscle isometric contraction, 15-min intermittent high-frequency electrical stimulation was used. Series consisted of trains of 2-ms rectangular pulses at a rate of 50 s^−1^ at 3 s duration and separated by 6 s intervals of rest. The stimulus current was set to 1.3–1.5 times the motor threshold. The signals (stimulus pulses, muscle tension and other) were sampled via DAC-ADC device (CED Power 1401, Cambridge Electronic Design, Cambridge, United Kingdom).

### 4.4. Data Analysis

For the statistical analyses, mechanograms of electrical muscle stimulation were divided into three phases in relation to the rate of decrease in muscle force contraction (0–100 s, 101–200 s and 201–900 s), which were normalized (in relation to the first three amplitudes peaks) and averaged. Thereafter, the parameters of muscle tension mechanogram such as the decay time constant (τ) and the amplitude of the process (A) were determined according to the formula: y = y_0_ + Ae^−x/τ^. Mean values (mean ± SD) of the TS muscle strength (each phases) of control mice, PN and PN+AD-MSCs animals were compared using a two-way statistical analysis of variance (ANOVA). The factors of variation included two conditions, group of animals and stimulation phases. A Bonferroni *post hoc* analysis was used to determine the differences between values. The level of significance was set at *p* < 0.05. Data analysis was performed using Spike 2 (Cambridge Electronic Design, Cambridge, United Kingdom) and Origin 8.5 (OriginLab Corporation, Northampton, MA, USA) software.

## 5. Conclusions

Thus, adipose-derived mesenchymal stromal cells treatment in mice with hereditary peripheral neuropathy resulted in a twofold increase in muscle contraction strength compared to untreated animals in this study. Although the recovery of the strength of muscle contraction was not complete, compared with control, however, this founding illustrates the effectiveness of AD-MSCs therapy for treatment peripheral neuropathies, in particular CMT1A. The main advantage of adipose-derived mesenchymal stromal cells over cells derived from other sources is that they can be easily and repeatable harvested in large quantities using minimally invasive techniques with low morbidity. We suggest that obtained data will help find new approaches in the treatment of such type diseases.

## Data Availability

The datasets generated during the current study are available from the corresponding author on reasonable request.
